# Supplementary material for: Intra-Tumoral CD8+:CD3+ Lymphocyte Density Ratio in Appendix Cancer Is a Tumor Volume- and Grade-Independent Predictor of Survival
Source: Cancers (Basel). 2025 Feb 6;17(3):542. doi: 10.3390/cancers17030542 (PMC11817446; doi:10.3390/cancers17030542)
Supplement: Supplementary file 1 [file cancers-17-00542-s001.zip › cancers-3401551-Table S1.pdf]

Supplementary Table S1. Subgroup comparisons of lymphocyte density parameters by histologic subtype. LAMN, low-grade appendiceal mucinous neoplasm; mAC, mucinous adenocarcinoma; nmAC, non-mucinous adenocarcinoma.

	LAMN vs. non-LAMN (mAC+nmAC)			Mucinous vs. non-mucinous			LAMN vs. mucinous AC vs. non-mucinous AC					
		median [IQR]	p		median [IQR]	p		median [IQR]	p, LAMN vs. mAC	p, LAMN vs nmAC	p, mAC vs. nmAC	p, trend
CD3+	LAMN	313.2 [192.9, 593]	0.009	Mucinous	310.9 [171.1, 484.9]	0.09	LAMN	589.3 [374, 1243.1]	0.026	0.017	0.52	0.01
	mAC+nmAC	589.3 [374, 1243.1]		nmAC	448.3 [259.8, 773.4]		mAC	333.5 [206, 645.7]				
							nmAC	310.9 [171.1, 484.9]				
CD8+	LAMN	199.4 [92.2, 386.5]	0.099	Mucinous	198.5 [92.2, 348.8]	0.16	LAMN	287.9 [152.2, 716.3]	0.27	0.07	0.5	0.08
	mAC+nmAC	287.9 [152.2, 716.3]		nmAC	232.8 [105.2, 636]		mAC	199.4 [95.7, 422.8]				
							nmAC	198.5 [92.2, 348.8]				
I-score	LAMN	45.2 [26.6, 66.5]	0.026	Mucinous	36.7 [26.6, 59]	0.1	LAMN	62.7 [34, 87.7]	0.096	0.024	0.47	0.02
	mAC+nmAC	62.7 [34, 87.7]		nmAC	52.1 [31.3, 80.3]		mAC	48.4 [31.3, 66.5]				
							nmAC	36.7 [26.6, 59]				
CD8+:CD3+ ratio	LAMN	0.59 [0.42, 1.11]	0.44	Mucinous	0.55 [0.41, 0.92]	0.77	LAMN	0.56 [0.36, 0.74]	0.3	0.73	0.46	0.82
	mAC+nmAC	0.56 [0.36, 0.74]		nmAC	0.58 [0.37, 0.99]		mAC	0.6 [0.52, 1.13]				
							nmAC	0.55 [0.41, 0.92]				
