# Supplementary material for: Intra-Tumoral CD8+:CD3+ Lymphocyte Density Ratio in Appendix Cancer Is a Tumor Volume- and Grade-Independent Predictor of Survival
Source: Cancers (Basel). 2025 Feb 6;17(3):542. doi: 10.3390/cancers17030542 (PMC11817446; doi:10.3390/cancers17030542)

Supplementary Figure S1. CD3^{+} or CD8^{+} cell density inverse association that appeared to be most prominent in patients above age 40 (as depicted in the top figure), male patients, in primary vs. metastatic lesions, in mucinous AC vs. other histologic types (as depicted in the lower figure), and in high- vs. low- grade tumors.

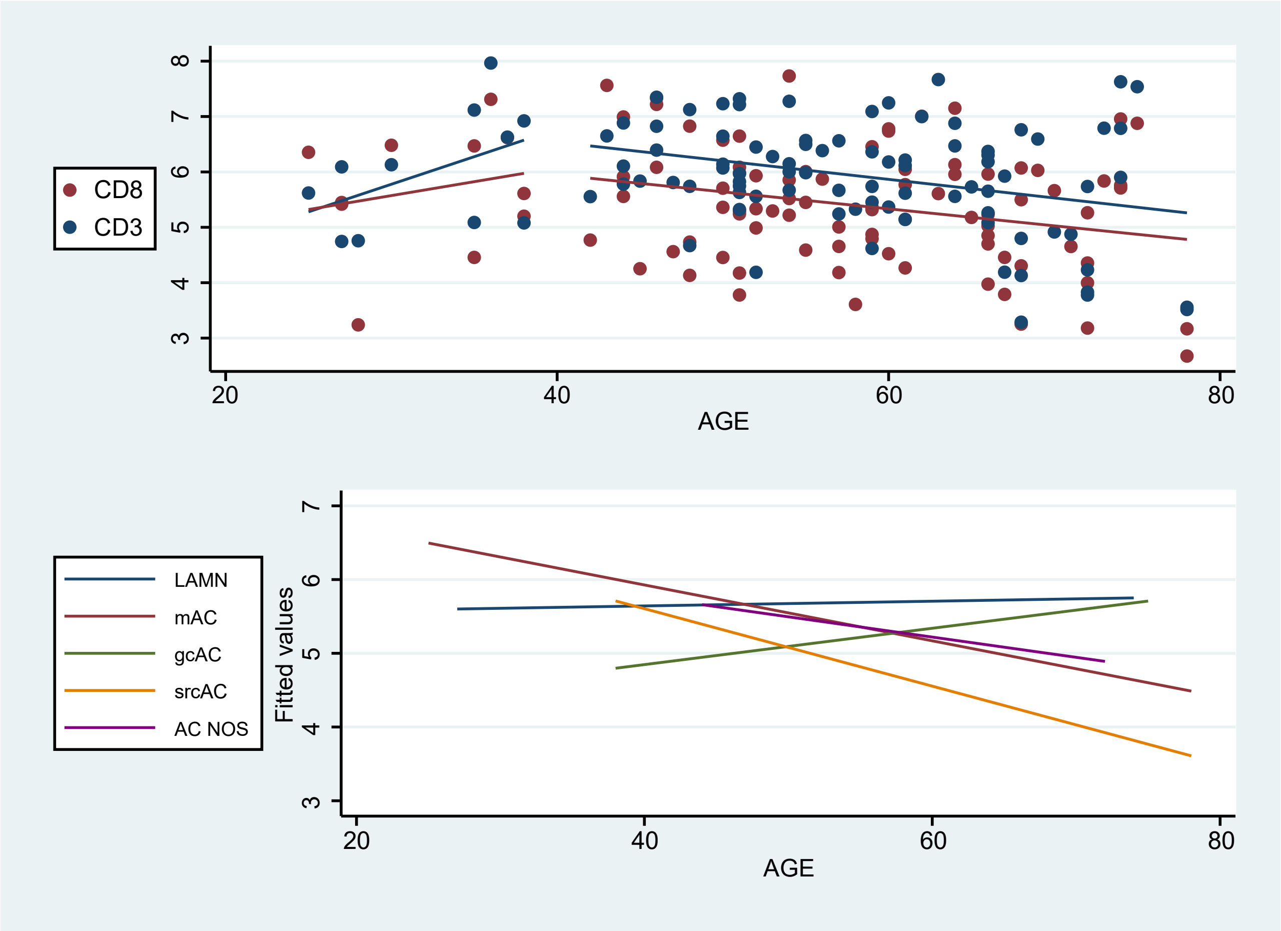

Supplementary Figure S2. Subgroup comparisons of lymphocyte density parameters by histologic subtype. LAMN, low-grade appendiceal mucinous neoplasm; mAC, mucinous adenocarcinoma; nmAC, non-mucinous adenocarcinoma.

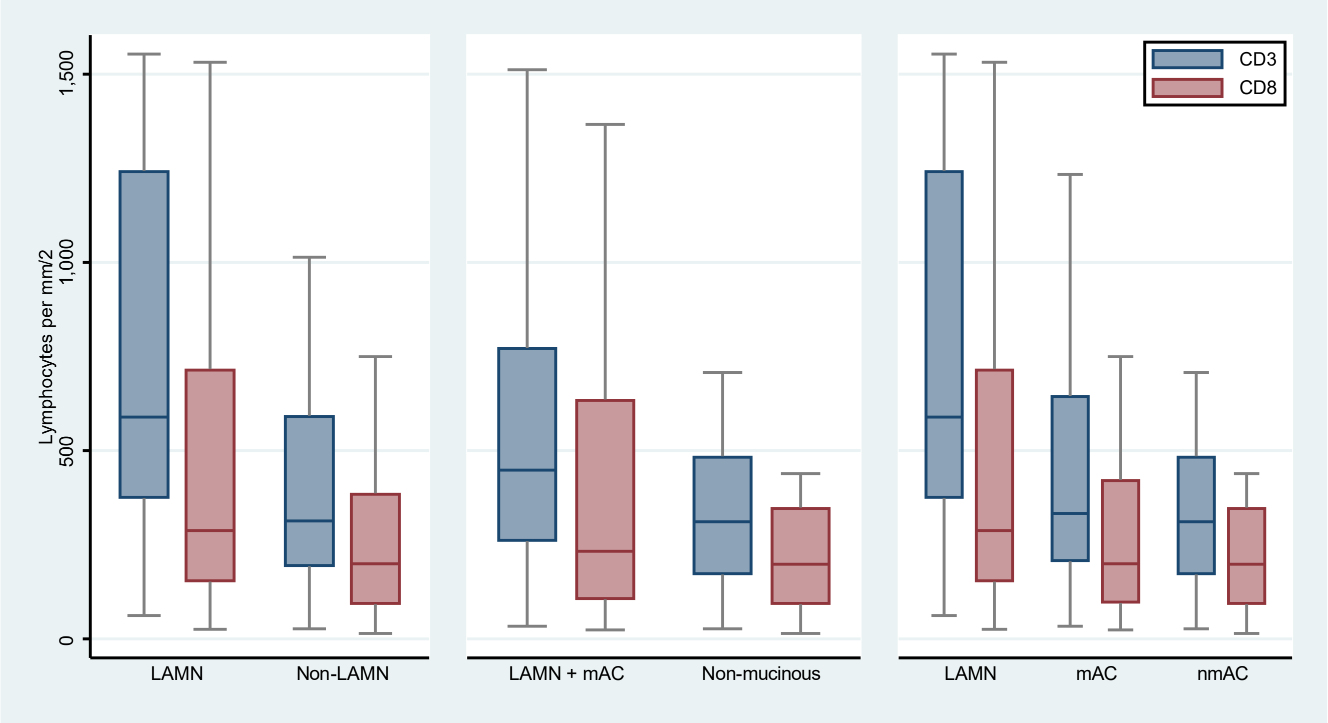

Supplementary Figure S3. Comparison of CD8+ density to AJCC stage, PCI and CEA level. PCI was found to have a negative association in LAMN (ρ = -0.5, *p* = 0.04) and AC-NOS (ρ = -0.7, *p* = 0.07), as opposed to a positive association between these variables in signet ring cell AC (ρ = 0.9, *p* = 0.05).

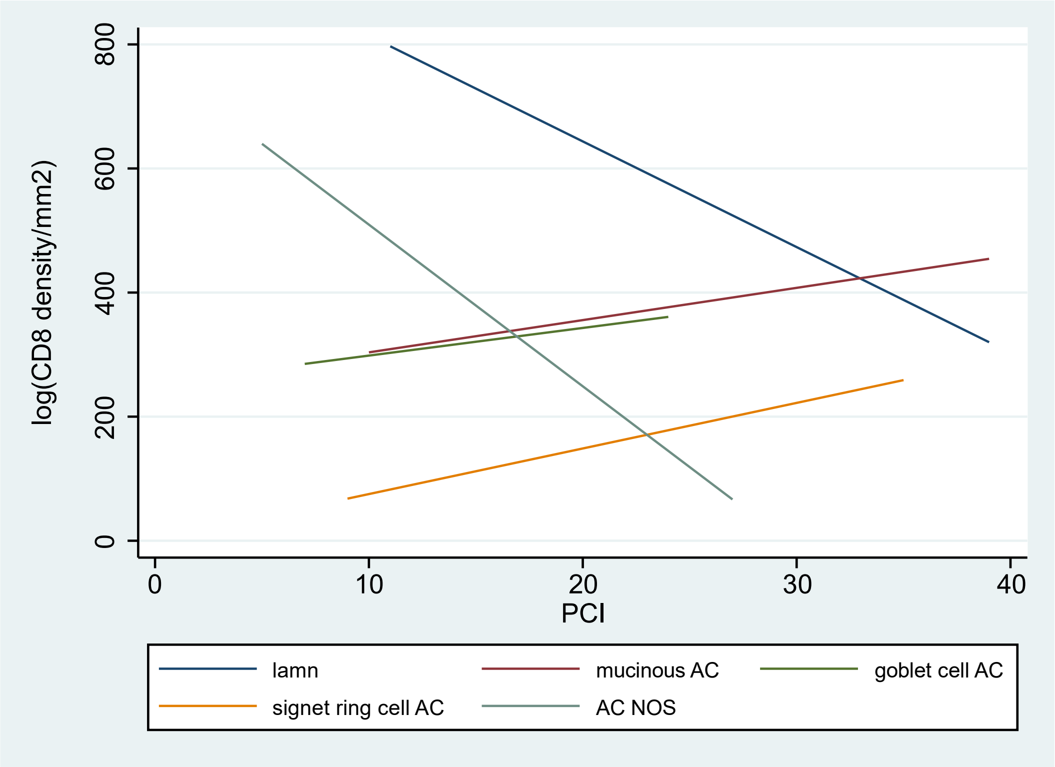

Supplementary Figure S4. Comparison of progression-free survival (PFS) by I-score in low-grade appendiceal neoplasms (Left) vs. Grade II/III cancers (Right). A discriminatory cutoff based on underlying CD3+ or CD8+ cell density could not be identified in this dataset, although PFS in low grade AC was associated with tumor I-score >70th percentile (HR 0.4, 95%CI [0.2, 1.0], *p* = 0.04)

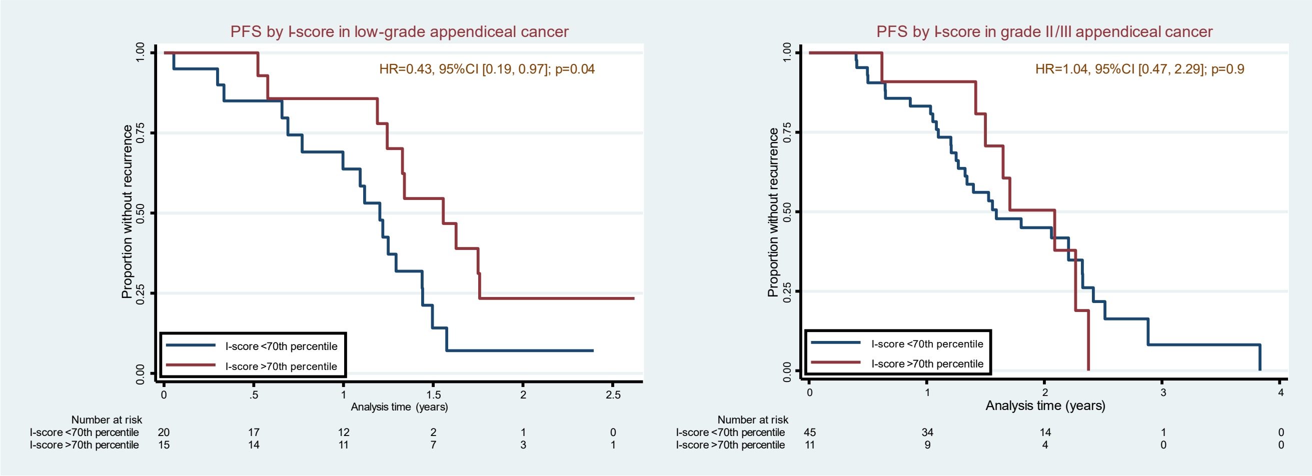

Supplement: Supplementary file 1 [file cancers-17-00542-s001.zip › Supplementary Figures.pdf]
